# 598 Reconstruction of Trochanteric Pressure Ulcers with Pedicled Vastus Lateralis-Anterolateral Thigh Flap

**DOI:** 10.1093/jbcr/irac012.226

**Published:** 2022-03-23

**Authors:** Burak Ozkan, Cagri A Uysal, Cem Aydogan, Mehmet Haberal

**Affiliations:** Baskent University, Ankara, Ankara; Baskent University Faculty of Medicine, Ankara, Ankara; Baskent University, Ankara, Ankara; Baskent University, Ankara, Ankara

## Abstract

**Introduction:**

Trochanteric region is one of the most frequent site for pressure ulcer development. High-voltage electric injuries with neurovascular damage is an important reason of paraplegia. These patients have prolonged hospitalization period and develop cachexia by time. Grade 3-4 sores with thoracanter major of femur exposition generally need flap reconstruction. Musculocutaneous flaps are preferred to cover exposed bone and fill the dead space. Although loco regional flaps such as tensor fasia lata, gluteus Maximus muscle flap have been used for thoracanteric ulcers, these flaps have mandatory muscles for ambulation in the following rehabilitation period. Another disadvantage of these flaps are the possible donor site problems due to pressure loading. Thus, we aimed to used vastus lateralis- anterolateral thigh(VL-ALT) flap for decreasing donor-site morbidity and tension.

**Methods:**

Between 2019 and 2021 we have operated 6 trochanteric pressure ulcers in 5 patients. All patients had history of electric injury related paraplegia. The mean age of the patients was 33 (24-50) The defect size were ranging between 5x4cm and 10x12 cm. Flap size were planned according to exact size of the defect. VL muscle were harvested according to the size of dead space. The donor site was closed primarily in 3 patients. Split thickness skin graft were needed in 2 patients. Patients were hospitalized in lateral decubitus or supine position during follow –up.

**Results:**

Mean follow up time was 10 months (6-18). All flaps were survived. Hematoma development were seen in 1 patient in the donor site which were treated with bedside debridement. No recurrence was seen during follow-up period. No restriction or morbidity were encountered during ambulation.

**Conclusions:**

VL-ALT musculocutaneous flap provides the required tissue for dead space filling and defect closing simultaneously. The advantages of this flap are; lower donor site morbidity, perfect match in terms of skin quality and bulk, protecting the major muscles such as gluteus maximus and tensor fascia lata. The technique needs knowledge of quadratus femoris anatomy which has a short learning curve.

